# Genomic insight into the common carp (*Cyprinus carpio*) genome by sequencing analysis of BAC-end sequences

**DOI:** 10.1186/1471-2164-12-188

**Published:** 2011-04-14

**Authors:** Peng Xu, Jiongtang Li, Yan Li, Runzi Cui, Jintu Wang, Jian Wang, Yan Zhang, Zixia Zhao, Xiaowen Sun

**Affiliations:** 1 The Centre for Applied Aquatic Genomics, Chinese Academy of Fishery Sciences, Beijing, 100141, China; 2 College of Fisheries and Life Science, Shanghai Ocean University, Shanghai, 201306, China; 3 College of Life Sciences, Tianjin Normal University, Tianjin, 300387, China

## Abstract

**Background:**

Common carp is one of the most important aquaculture teleost fish in the world. Common carp and other closely related Cyprinidae species provide over 30% aquaculture production in the world. However, common carp genomic resources are still relatively underdeveloped. BAC end sequences (BES) are important resources for genome research on BAC-anchored genetic marker development, linkage map and physical map integration, and whole genome sequence assembling and scaffolding.

**Result:**

To develop such valuable resources in common carp (*Cyprinus carpio*), a total of 40,224 BAC clones were sequenced on both ends, generating 65,720 clean BES with an average read length of 647 bp after sequence processing, representing 42,522,168 bp or 2.5% of common carp genome. The first survey of common carp genome was conducted with various bioinformatics tools. The common carp genome contains over 17.3% of repetitive elements with GC content of 36.8% and 518 transposon ORFs. To identify and develop BAC-anchored microsatellite markers, a total of 13,581 microsatellites were detected from 10,355 BES. The coding region of 7,127 genes were recognized from 9,443 BES on 7,453 BACs, with 1,990 BACs have genes on both ends. To evaluate the similarity to the genome of closely related zebrafish, BES of common carp were aligned against zebrafish genome. A total of 39,335 BES of common carp have conserved homologs on zebrafish genome which demonstrated the high similarity between zebrafish and common carp genomes, indicating the feasibility of comparative mapping between zebrafish and common carp once we have physical map of common carp.

**Conclusion:**

BAC end sequences are great resources for the first genome wide survey of common carp. The repetitive DNA was estimated to be approximate 28% of common carp genome, indicating the higher complexity of the genome. Comparative analysis had mapped around 40,000 BES to zebrafish genome and established over 3,100 microsyntenies, covering over 50% of the zebrafish genome. BES of common carp are tremendous tools for comparative mapping between the two closely related species, zebrafish and common carp, which should facilitate both structural and functional genome analysis in common carp.

## Background

Cyprininae carps are the most important cultured species, accounting for over 30% aquaculture production in the world. Common carp (*Cyprinus carpio*) is currently one of the top three cultured carps in China. Because of its importance, genetic studies have been conducted in the last several decades for cellular and molecular components of the carp genome. The common carp genome is composed of 100 chromosomes. It has been believed to be a tetroploid species with a physical size of approximately 1700 Mbp (2n).

Teleosts are widely believed to have gone through an additional round of whole genome duplication, i.e., the 3R hypothesis, as compared to mammals. Common carp is believed to have had another round of genome duplication (4R) and became a evolutionarily recent tetraploid fish [1]. As such, it is widely used as model species for evolutionary studies such as fish specific genome duplication, gene loss after whole genome duplication, and functional partitioning of duplicated genes [2-4]. Much research efforts have been made for the understanding of the carp genome including development of polymorphic markers [5-7], linkage mapping [8,9], and quantitative trait loci (QTL) analysis [10,11]. However, such research has been limited by the lack of large-scale genomic resources.

Analysis of BES has proven to be an effective approach for development of markers that are not only useful for linkage mapping, but for the integration of genetic linkage and physical maps [12,13]. In teleost fish, a large set of BES data had been developed in several economically important speices, including catfish [13,14], rainbow trout [15,16], Atlantic salmon [17], tilapia [18] and European sea bass [19]. In order to provide initial insight into the carp genome and generate a large number of polymorphic markers for genetic and genomic analysis, and also to assess the repeat structure of the carp genome to provide information for whole genome sequencing and provide paired reads of large genomic clones for the whole genome assembly [13,19-23], here we report the generation and analysis of 80,000 BAC end sequences (BES).

## Result and Discussion

### Generation of BAC-end sequences

**Figure 1:**
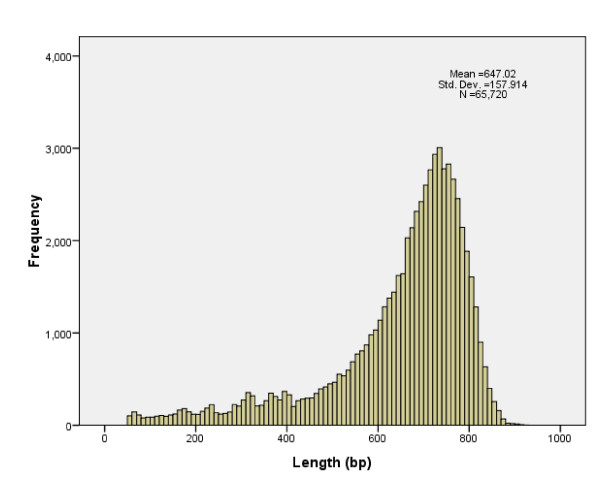
**Read length distribution of BES after base calling, trimming for *E. coli *and vector sequences and removing redundancies**.

A total of 40,224 BAC clones, representing 3.34X clonal coverage of the common carp genome, were sequenced from both ends. There were 75,744 BES with minimum length of 50 bp. After base calling and trimming for *E. coli *and vector sequences, 72,789 (96.1% success rate) high quality (Q20) BES with minimum length of 50 bp were generated. Further, 7,069 redundant BES with 95% identity and full-length covered were removed. The remained 65,720 BES are total 42,522,168 bp in length, representing approximately 2.5% of the common carp genome (Table 1). The lengths of BES ranged from 50 to 924 bp, with an average length of 647 bp (Figure 1). Of these 65,720 BES, 29,046 BAC clones (88.4%) were successfully sequenced on both ends, generating mate-pair reads. Sequence analysis of the BES indicated that the carp genome, like many other teleost genomes, is A/T-rich with 63.2% A/T and 36.8% G/C. The BES sequences were deposited into GenBank with continuous accession numbers of HN150714-HN153235 and HR505563-HR575920.

**Table 1:** Sequence statistics of the BES of common carp

BAC end sequencing reads	80,448
BES ^a^	75,744

BES after trimming ^b^	72,789(96.1%)

Redundant BES ^c^	7,069

BES after filtering redudance	65,720

Average read length (bp)	647

BES mate-pairs ^d^	29,046(88.4%)

Total bases sequenced	42,522,168

Vertebrates Repeat masked bases	7,356,797(17.3%)

^a ^Number of sequences with phred base calling (quality score ≥ 20) with edited read length ≥50 bp

^b ^Number of sequences free of *E. coli *and vector DNA, with edited read length ≥50 bp

^c ^Number of sequences with 95% identity and full-length covered by selfblasting

^d ^Number of clones with both ends successfully sequenced

### Assessment of the repetitive elements in the carp genome

The proportion of the repetitive elements in the common carp genome was assessed by using RepeatMasker[24] with *Vertebrates *Repeat Database. Repeatmasking of the 42,522,168 bp of the carp BES sequences resulted in the detection of 7,357,899 (17.3%) base pairs of repeated sequences. The classification and respective proportion of the identified repetitive elements are shown in Additional File 1. The most abundant type of repetitive element in the common carp genome was DNA transposons (6.67%), mostly hobo-Activator (2.25%), followed by retroelements (4.52%) including LINEs (2.33%), LTR elements (1.98%), and SINEs (0.2%). Various satellite sequences, low complexity and simple sequence repeats accounted for 2.46%, 1.98% and 1.64% of the base pairs, respectively. The repeats divergence rate of DNA transposons (percentage of substitutions in the matching region compared with consensus repeats in constructed libraries) showed a nearly normal distribution with a peak at 24%. A fraction of LTR retrotransposons, LINEs and SINEs had nearly the same divergence rates as DNA transposons (peaks at 30%, 28% and 22%, respectively), indicating relatively old origin (Additional File 2). Additional 518 BES that had not been masked by RepeatMasker were identified as homologs of proteins encoded by diverse families of transposable elements using transposonPSI[25].

To identify novel repetitive elements in the common carp genome, repeat libraries were constructed using multiple *de novo *methods and then combined into a non-redundant repeat library containing 1,940 sequences. The repeat library was then used for repeat annotation of the common carp BES. Additional total of 4,499,836 bp were identified, representing approximately 10.6% of the BES, as *de novo *repeats.

### Identification of microsatellites from BES

**Figure 2:**
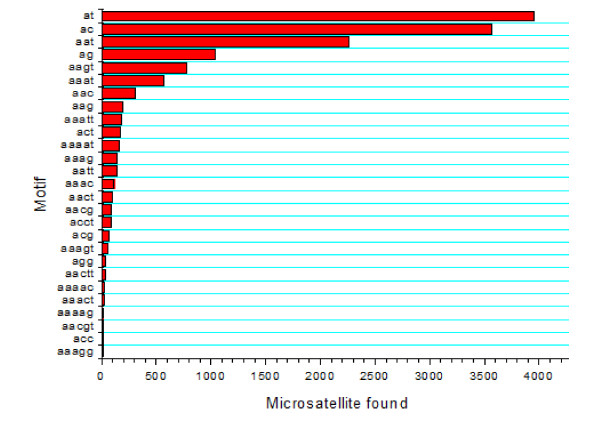
**Distribution of major microsatellite types in common carp BES**.

From the 65,720 common carp BES, 10,355 BES were found to contain microsatellites with a total of 13,581 microsatellites. The vast majority of the BES-associated microsatellites were di-nucleotide repeats (8,126, 59.83%), followed by tri-nucleotide repeats (2,927, 21.55%), tetra-nucleotide repeats (1,950, 14.36%), penta-nucleotide repeats (549, 4.04%), and hexa-nucleotide repeats (just 29). As shown in Figure 2, AT motif was the most abundant type of microsatellites, followed by motifs of AC, AAT, AG and AAGT, whereas GC-rich motif was very low. An analysis of flanking sequences indicated that of these identified microsatellites, 5,150 had sufficient flanking sequences for PCR primer design.

### Identification of protein-coding sequences and functional annotation

After repeat and transposon ORFs masking, 65,202 BES had greater than 50 bp of contiguous non-repetitive sequences. Protein-coding sequences were identified by homology searches with BLASTX against non-redundant protein database. A total of 9,443 BES had significant hits at the e-value cutoff of e^-5 ^with 7,127 distinct gene hits. As expected, the vast majority, 5,146 (72.2%) of the best hits were zebrafish genes, indicating high levels of sequence similarity between the zebrafish and carp genomes.

### Anchoring of carp BES to the Zebrafish Genome

Zebrafish is the most closely related species to common carp among teleost fishes with a draft whole genome sequence. They both belong to the same family of Cyprinidae. A large set of BES from common carp generated from this study allowed the possibility to conduct initial comparative genome analysis between zebrafish and common carp. In order to map common carp BES to zebrafish chromosomes, BLASTN searches of the common carp BES against zebrafish zv8 assembly were conducted, which resulted in significant hits (e-5 cutoff) by 39,335 query BES, of which 16,267 had unique hits to the zebrafish genome. The ratio of unique hits was much lower than that in cattle-human comparative analysis [26], which indicate that many BES of common carp have more than one homolog in zebrafish genome, implying the genome duplication status of Cyprinidae fish.

**Figure 3:**
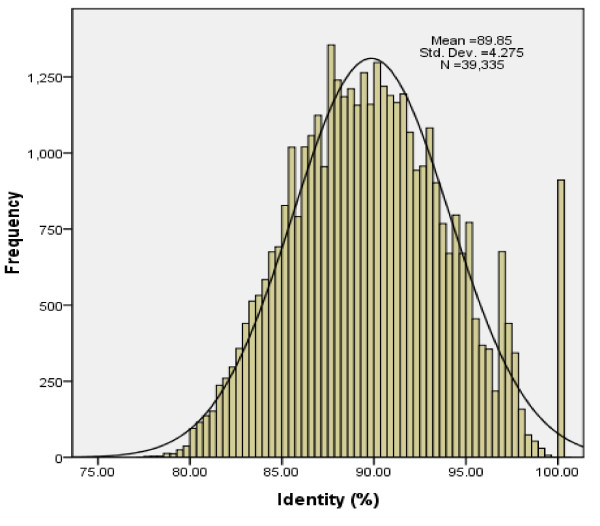
**Distribution of identity scores of the carp-BES alignments to zebrafish genome assembly 8 (zv8)**. Frequencies of BES with percent identity scores from 75 to 100% are shown.

The top alignment hits were selected to calculate the difference between the common carp and zebrafish genomes at the nucleotide level. The number of sites in top alignments for 39,335 query BES were 6,773,762, of which 6,120,195 sites were identical to their zebrafish counterparts for a mean percent identity of 90.4%. The distribution of the percent identity of BES is depicted in Figure 3. The distribution is nearly normal distribution with a mean of the difference at around 10%. However, there was a burst of BES enriched in 100% identity in the genome, indicating the most conserved elements shared by carp and zebrafish.

Using annotated protein-coding gene regions in the zebrafish genome, we found that carp BES located in exon regions of 5,857 zebrafish protein-coding genes, which are much more than the number of 5,146 zebrafish genes identified from NR database with BLASTX method as we reported above. Mostly likely, some of BES might be homolog to the UTR regions of zebrafish genes which could not identify zebrafish coding regions from protein NR database.

To construct a zebrafish-carp comparative map, additional analyses were focused on paired BAC clones with top hits on both ends. Among 29,046 BAC clones with BES mate-pairs, 26,809 had both ends matching zebrafish genome sequence, of which 3,133 had ends ≤ 300 kb apart on the same chromosome and with the same orientation (Table 2). After summing the physical coverage on zebrafish, we found that there were 50.77% of zebrafish genome covered by the carp BACs (Table 3), indicating the high similarity of these two genomes. Apparently, the largest number of conserved microsyntenies was constructed on zebrafish chromosome 7, followed by chromosome 9, chromosome 4, chromosome 5 and chromosome 17. Chromosome 25 had the smallest number (51) of conserved microsyntenies with common carp. The microsyntenies on chromosome 17 had the largest coverage (70.92%). Conserved microsyntenies were then divided into five categories: 1) microsyntenies with both ends being protein-coding genes (including introns); 2) microsyntenies with one end being a conserved protein-coding gene, and the other end being conserved non-coding gene; 3) microsyntenies with both ends being conserved non-coding gene; 4) microsyntenies with one end being a conserved protein-coding gene while the other end being a putative intergenic region; and 5) microsyntenis with one end being a conserved non-coding gene while the other end being a putative intergenic region. As summarized in Table 4, 697 type 1, 4 type 2, and 13 type 3 conserved microsyntenies were identified. While the confidence for conserved microsyntenies of type 3 and type 5 is not high, microsyntenies with similar spacing on the genomes of both common carp and zebrafish strongly suggest that those regions are real conserved syntenic regions, which should be valuable resource for comparative mapping and evolutionary studies.

**Table 2:** Summary of BES mapping

		Number of BAC clones	Number of BES
Mate-pairs		29,046	

Mapped		26,809	38,736

	Paired ends^a^	3,133	6,266

	Long BES pairs^b^	3,182	6,364

	mate-reverse^c^	267	534

	Unmate^d^	5,345	10,690

	Singleton^e^	14,882	14,882

Unmapped		2,237	4,474

a. Two ends (≤ 300 kb apart) derived from the same BAC clone were mapped to the same chromosome with the same orientation.

b. Two BES (>300 kb apart) in one BAC located in the same chromosome with the same orientation.

c. Two BES in one BAC located in the same chromosome with the different orientation.

d. Two ends from the same BAC were mapped to different chromosomes.

e. Only one end in carp BAC was aligned to zebrafish chromosome.

**Table 3:** Estimated coverage of chromosomes by the common carp BACs. Zebrafish genome assembly 8 (zv8) were used for the calculation.

Chromosome	Size (bp)	Covered (bp)	Number of clones*	Coverage
1	59,305,620	28,385,015	132	47.86%

2	58,009,534	22,677,316	102	39.09%

3	60,907,308	31,396,678	148	51.55%

4	71,658,100	32,427,816	168	45.25%

5	74,451,498	35,726,625	164	47.99%

6	61,647,013	32,747,166	149	53.12%

7	76,918,211	46,016,985	220	59.83%

8	55,568,185	27,732,046	123	49.91%

9	54,736,511	37,568,203	176	68.63%

10	43,467,561	18,729,705	83	43.09%

11	44,116,856	24,200,149	108	54.85%

12	46,853,116	23,019,149	102	49.13%

13	50,748,729	33,185,367	153	65.39%

14	52,930,158	21,972,192	105	41.51%

15	47,237,297	23,826,254	111	50.44%

16	51,890,894	22,116,433	100	42.62%

17	49,469,313	35,083,736	161	70.92%

18	49,271,716	24,262,639	113	49.24%

19	48,708,673	19,679,618	87	40.40%

20	51,884,995	34,768,791	151	67.01%

21	47,572,505	18,049,644	89	37.94%

22	41,415,389	20,094,889	100	48.52%

23	44,714,728	24,485,475	115	54.76%

24	40,403,431	22,721,787	106	56.24%

25	38,768,535	10,635,174	51	27.43%

Total	1,322,655,876	671,508,852	3,117	50.77%

*BAC clones are counted only when they have two sequenced ends in the same chromosome with the correct orientation.

**Table 4:** Five categories of conserved microsyntenies

Type	Number of BAC clones
Type1 (protein-coding *vs *protein-coding)	697

Type2 (protein-coding *vs *non-coding)	4

Type3 (non-coding *vs *non-coding)	13

Type4 (protein-coding *vs *intergenic)	2,025

Type5 (non-coding *vs *intergenic)	4

## Conclusion

BAC end sequences were important resource for many genomic studies, especially for the whole genome sequencing and assembly of a large and complex genome. To better understanding of common carp genome, the large scale BAC end sequencing had been conducted on over 40,000 BAC clones. The first survey of common carp genome and the first genome wide comparative analysis of common carp and zebrafish genomes had been accomplished.

The information of repetitive elements in the carp genome is eager to know for upcoming whole genome sequencing and genome assembly. Multiple bioinformatic approaches had been employed and the known repetitive DNA similar to vertebrates was estimated to be approximate 17.3% of common carp genome, which is lower than another tetraploid teleost fish Atlantic salmon (30-35%) [27], but higher than catfish [14].

A total of 7,127 distinct homolog genes had been identified from surveyed BES of common carp. The vast majority were zebrafish genes, suggesting the high similarity of the zebrafish and carp genomes. Further comparative analysis mapped around 40,000 BES to zebrafish genome. With mate-paired BES, over 3100 microsyntenies had been constructed between common carp and zebrafish genome, covering over 50% of the zebrafish genome. As parts of "Common Carp Genome Project", both fingerprint-based physical map and high-density linkage map of common carp genome are ongoing and the completion is expected in 2011. Once the two maps are available, these BES and microsyntenies will be valuable resource to construct the genome scale zebrafish-common carp fine comparative map for the whole genome assembly and important traits localization of common carp.

## Methods

### BAC library

The common carp BAC library, constructed with genomic DNA from a female individual, containing 92,160 BAC clones with an average insert size of 141 kb, was used for generating BAC-end sequences [28].

### BAC Culture and End Sequencing

BAC clones were inoculated into deep 96-well culturing blocks containing 1.2 ml 2 × YT medium and 12.5 μg/ml chloramphenicol from 384-well stocking plates using 96-pin replicator (V&P Scientific, Inc., San Diego, CA). The culture blocks were sealed with an air permeable seal (Excel Scientific, Wrightwood, CA) and shaked at 37°C for 20 hours with the speed of 300 rpm. The bacteria were then collected by centrifugation at 2000 g for 10 min in a Beckman Avanti J-26 XP centrifuge. After carefully removing all liquid from the culture blocks, bacterial pellets were used for BAC DNA extraction by using an alkaline lysis protocol [29] with modification on lysate clarification. The fritted filter plates (NUNC, Roskilde, Denmark) were used for lysate filtration, which significantly increased the BAC DNA quality for BAC end sequencing. BAC DNA was precipitated with isopropanol and washed with 70% ethanol twice. BAC DNA was then eluted into 40 μl milliQ water and collected in 96 plates and stored in -20°C before use.

Sanger sequencing reactions were conducted in 96-well semi-skirt plates using the following ingredients: 2 μl 5X Sequencing Buffer, 2 μl sequencing primer (3 pmol/μl), 1 μl BigDye v3.1 Dye Terminator(Life Technology, Foster City, CA), and 5 μl BAC DNA. The sequencing reactions were conducted in ABI 9700 Thermal Cyclers (Life Technology) under the following conditions: initial 95°C for 5 min; then 99 cycles of 95°C for 30 sec, 55°C for 10 sec, 60°C for 4 min. The T7 and PIBRP primers were used for sequencing reactions (T7 primer: TAATACGACTCACTATAGGG; PIBRP primer: CTCGTATGTTGTGTGGAATTGTGAGC). The sequencing reactions were then precipitated with pre-chilled 100% ethanol and cleaned up with 70% ethanol. The samples were then analyzed with ABI 3730 XL (Life Technology).

### Clone Tracking and Quality Control

In order to avoid any orientation mistake, eight clones were re-sequenced from each 384-plate from positions A1, A2, B1, B2, C1, C2, D1, and D2. The quality control sequences were then searched against all collected BAC end sequences with BLAST program. The re-sequencing data hit the BES with a same well position will assure the correct plate orientation.

### Sequence Processing

The software Phred [30,31] was used for the BAC end sequences base calling. Quality score of Q20 was used as a cutoff in base calling. Seqclean [32] in DFCI Gene Indices Software Tools was used for vector trimming against UniVec database [33] with default parameter values. The trimmed BES were searched against themselves with BLASTN and BES that have >95% identity with other BES and have full-length covered in the alignment were filtered out in the following analysis.

### Repeat analysis

To detect known repeats in carp BES, we screened and masked BES using Repeatmasker software [24] againt *Vertebrates *Repeat library with default parameter values. Next, BES homology to proteins encoded by diverse families of transposable elements were searched using TransposonPSI [14], a program that performs tBLASTn searches using a set of position specific scoring matrices (PSSMs) specific for different transposon element families.

Two *de novo *software packages, PILER-DF [34] and RepeatScout [35], were used to search for *de novo *repeat sequences within carp BES and built two repeat libraries, respectively. The repeat sequences in one library were compared with those sequences in the other one using BLASTN. The shorter sequences were filtered when two repeats aligned with identity ≥ 95% and coverage ≥ 95% of full length. A non-redundant *de novo *repeat library of common carp was then constructed with those distinct repeat sequences. The BES that were neither masked with known vertebrates repeat library nor similar to TE, were then searched against the *de novo *repeat library with RepeatMasker.

### Identification of Microsatellites

Microsatellites were identified in non-redundant BES by using the perl script Msatfinder which was specifically designed to identify and characterize microsatellites[36]. Only the microsatellites of 2-6 nucleotide motifs with at least 5 repeat units were collected.

### Gene prediction

BLASTX searches of the repeat-masked BES were conducted against the Non-Redundant Protein database. A cut off e-value of e^-5 ^was used as the significance similarity threshold for the comparison. The top BLASTX result of each BES query was collected.

### Comparative Genomics

To compare the similarity of common carp and zebrafish genomes and anchor common carp BACs to zebrafish genome, we assumed that the zebrafish genome assembly is correct and carp BES that were masked with repeats and transposons, were searched against zebrafish genome assembly 8 (zv8) by using the program BLASTN with e-value cutoff 10^-5^. The top hit of each BES were further analyzed.

The conserved microsyntenies were defined as the alignment regions where carp BAC clones had ends ≤ 300 kb apart on the same chromosome and with the same orientation. Conserved microsyntenies were then divided into five categories based on transcriptional signals in zebrafish homolog genome regions to carp BES. Zebrafish Refseq genes as transcriptional signals were downloaded from UCSC database [37] and divided into protein-coding genes and non-coding genes from their annotation.

## Authors' contributions

PX and JL contributed equally and their contribution accounts for the major part of this study. PX designed and supervised the BAC-end sequencing project, and drafted the whole manuscript. JL worked on bioinformatic analysis and participated in the manuscript revision. YL generated all BAC end sequences. RC participated in the BAC end sequencing. JTW and JW participated in BAC culture and DNA extraction. YZ participated in microsatellite identification. ZZ participated in BAC library duplication. XS supervised the common carp genome project. All authors read and approved the final manuscript.

## Supplementary Material

Additional file 1 **The repetitive elements in carp genome**. The file contains percentage of different *Vertebrates *repeats in carp genome, screened with RepeatMasker software. Click here for file

Additional file 2 **Repeat divergence in carp genome**. The file describes the sequence divergence distribution from four major types of *Vertebrates *repeats in carp genome. Click here for file
