# Supplementary material for: Genomic insight into the common carp (Cyprinus carpio) genome by sequencing analysis of BAC-end sequences
Source: BMC Genomics. 2011 Apr 14;12:188. doi: 10.1186/1471-2164-12-188 (PMC3083359; doi:10.1186/1471-2164-12-188)
Supplement: Additional file 1 — The repetitive elements in carp genome. The file contains percentage of different Vertebrates repeats in carp genome, screened with RepeatMasker software. [file 1471-2164-12-188-S1.DOC]

|  |  | number of elements | length occupied  (bp) | Percentage of  sequence |
| --- | --- | --- | --- | --- |
| Retroelements |  | 7,427 | 1,921,032 | 4.52% |
|  | SINEs | 1,021 | 87,069 | 0.20% |
|  | Penelope | 4 | 409 | 0.00% |
|  | LINEs | 3,615 | 991,222 | 2.33% |
|  | L2/CR1/Rex | 3,076 | 823,424 | 1.94% |
|  | R1/LOA/Jockey | 96 | 28,184 | 0.07% |
|  | R2/R4/NeSL | 3 | 621 | 0.00% |
|  | RTE/Bov-B | 81 | 22,963 | 0.05% |
|  | L1/CIN4 | 305 | 100,434 | 0.24% |
|  | LTR elements: | 2,791 | 842,741 | 1.98% |
|  | BEL/Pao | 241 | 109,874 | 0.26% |
|  | Ty1/Copia | 12 | 5,507 | 0.01% |
|  | Gypsy/DIRS1 | 1,865 | 594,130 | 1.40% |
|  | Retroviral | 350 | 53,128 | 0.12% |
|  |  |  |  |  |
| DNA transposons |  | 19,099 | 2,834,598 | 6.67% |
|  | hobo-Activator | 8,349 | 955,257 | 2.25% |
|  | Tc1-IS630-Pogo | 2,756 | 729,561 | 1.72% |
|  | En-Spm | 1,395 | 118,524 | 0.28% |
|  | MuDR-IS905 | 10 | 1,444 | 0.00% |
|  | PiggyBac | 785 | 169,329 | 0.40% |
|  | Tourist/Harbinger | 671 | 88,537 | 0.21% |
|  | Other(Mirage,  P-element, Transib) | 5 | 397 | 0.00% |
| Unclassified |  | 278 | 20,083 | 0.05% |
| Small RNA |  | 473 | 44,816 | 0.11% |
| Satellites |  | 5,522 | 1,044,099 | 2.46% |
| Simple repeats |  | 14,163 | 697,115 | 1.64% |
| Low complexity |  | 18,216 | 840,178 | 1.98% |
