# Supplementary figures and images for: Genomic insight into the common carp (Cyprinus carpio) genome by sequencing analysis of BAC-end sequences
Source: BMC Genomics. 2011 Apr 14;12:188. doi: 10.1186/1471-2164-12-188 (PMC3083359; doi:10.1186/1471-2164-12-188)

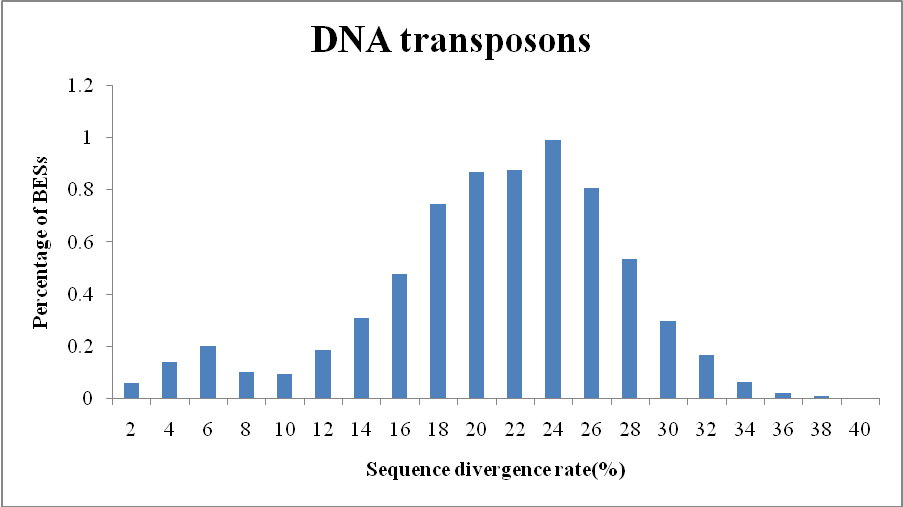

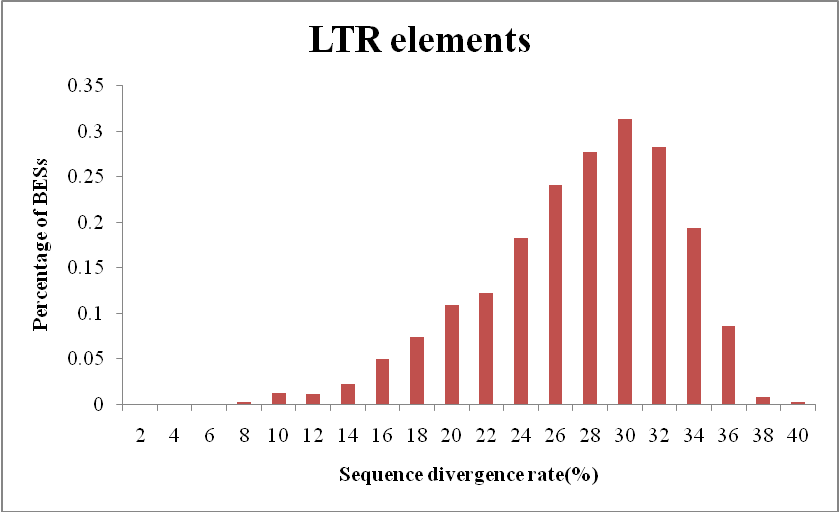

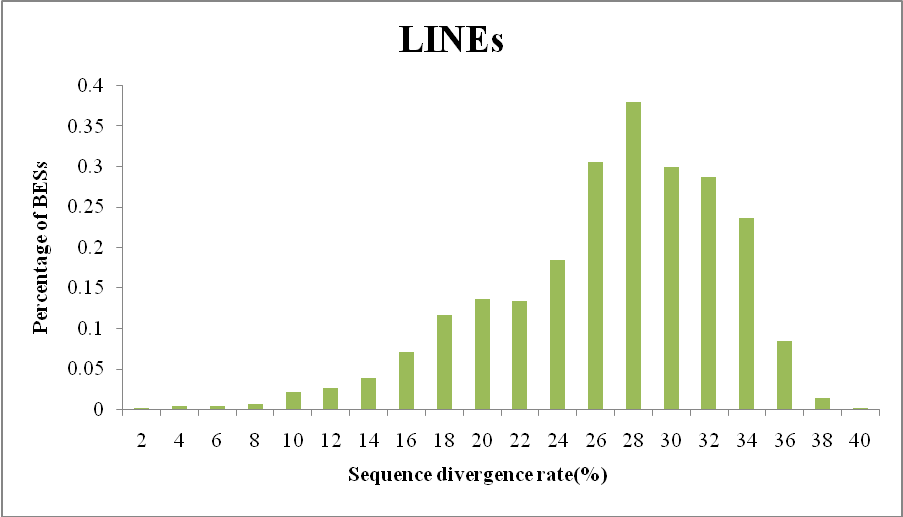

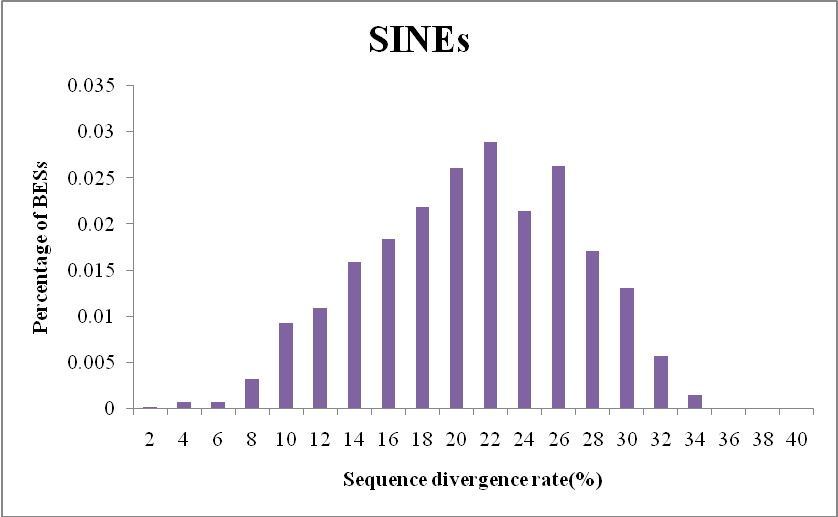

Supplement: Additional file 2 — Repeat divergence in carp genome. The file describes the sequence divergence distribution from four major types of Vertebrates repeats in carp genome. [file 1471-2164-12-188-S2.DOC]
